# A Rare Case of Purpura Fulminans in the Setting of Klebsiella pneumoniae Bacteremia

**DOI:** 10.7759/cureus.22921

**Published:** 2022-03-07

**Authors:** Ariel Ruiz de Villa, Kipson Charles, Peters Okonoboh

**Affiliations:** 1 Internal Medicine, University of Central Florida College of Medicine, Graduate Medical Education/North Florida Regional Medical Center, Gainesville, USA; 2 Internal Medicine - Critical Care, University of Central Florida College of Medicine, Graduate Medical Education/North Florida Regional Medical Center, Gainesville, USA

**Keywords:** critical care, klebsiella pneumoniae, disseminated intravascular coagulation, acute infectious purpura fulminans, purpura fulminans

## Abstract

A 23-year-old man with circulatory shock associated with severe sepsis and congestive heart failure with an ejection fraction of 10% resulting in anasarca and multiorgan failure was admitted to our hospital's intensive care unit. Hours after admission, he developed a rash on his left inner thigh, which was later diagnosed as purpura fulminans (PF). Blood cultures were consistent with* Klebsiella pneumoniae* bacteremia, with community-acquired pneumonia being the possible source.

PF is a rare and difficult-to-diagnose entity characterized by dysregulated hemostasis that is often associated with poor prognosis and fatal outcomes. To our knowledge, there are limited reports in the literature on *K*.* pneumoniae* as a cause of PF. Given the rarity of this presentation, this case will serve as an opportunity to report and discuss the pathophysiology of this disease for the benefit of physicians.

## Introduction

Purpura fulminans (PF) is considered a fatal thrombotic disorder that requires immediate diagnosis and management. It is rapidly progressive and often accompanied by disseminated intravascular coagulation and circulatory collapse. PF is associated with approximately 60% mortality among patients of all ages [1,2] and is typically observed in the setting of intrinsic coagulation disorders and complicated infections or idiopathically [3]. The affected patients are generally critically ill and managed in the intensive care unit (ICU). Its presentation often progresses within hours from regional erythema and petechiae evolving into purpuric plaques and ecchymoses, which can turn into gangrene and compartment syndrome if the patient does not succumb to the disease earlier.

When bacterial septicemia is a cause of PF, the most common bacterial cultures reported in the medical literature are gram-positive cocci such as *Neisseria meningitidis, Haemophilus influenzae,* and *Streptococcus pneumoniae* [3,4]. Among other unique and occurring cases described in published reports, to the best of our knowledge, only three other cases involved *Klebsiella pneumoniae*​​​​​​ [5]. Therefore, this case report on PF can be considered rare and complicated in the setting of *K. pneumoniae* bacteremia.

## Case presentation

A 23-year-old man with no reported past medical history presented to the emergency department with a one-week history of back, abdominal, upper and lower extremity, and testicular pain, associated with shortness of breath, severe weakness, and generalized swelling. He denied having fevers, chills, chest pain, palpitations, nausea, vomiting, headaches, and changes in urinary or bowel habits. He also denied having any pertinent family history, trauma, and recent animal or insect bites. He had a remote history of alcohol use but denied the use of illicit substances. On the way to the hospital via ambulance, the patient had atrial fibrillation and was treated with diltiazem and amiodarone, which resulted in a normal sinus rhythm. In the emergency department, his vital signs were as follows: pulse rate of 131 beats/min, respiration rate of 18 breaths/min, blood pressure of 109/57 mmHg, and 95% O2 saturation on pulse oximetry. The initial laboratory findings are shown in Table 1. Toxicology screening was negative, and urinalysis showed normal findings.

**Table 1:** Initial laboratory findings.

Laboratory test	Results
White blood cell count	9.3 × 10^3^/µL
Red blood cell count	5.09 × 10^6^/µL
Hemoglobin	14.7 g/dL
Hematocrit	41.9%
Platelets	145 × 10^3^/µL
Sodium	118 mmol/L
Potassium	5.2 mmol/L
Chloride	85 mmol/L
Magnesium	2.6 mg/dL
Phosphorus	4.70 mg/dL
Carbon dioxide	23 meq/L
Blood urea nitrogen	82 mmol/L
Creatinine	2.31 mmol/L
Glucose	94 mmol/L
Calcium	76 mmol/L
Total bilirubin	5 mmol/L
Lactic acid	7.3 mmol/L
Aspartate aminotransferase (AST)	258 U/L
Alanine aminotransferase (ALT)	543 U/L
Alkaline phosphatase	114 U/L
C-reactive protein	5.56 mmol/L
N-terminal pro-brain natriuretic peptide	43,561 pg/mL
Albumin	2.8 g/dL

Physical examination revealed that he was critically ill with grade 4+ edema of upper and lower extremities along with pronounced testicular edema. Cardiopulmonary examination revealed distant heart sounds, tachycardia, diminished peripheral pulses, and crackles on all lung fields. His abdomen was distended with a positive fluid wave. A nonconfluent and nontender petechial rash measuring approximately 6 × 4 inches was observed in the medial thigh. The patient had a normal sclera, moist mucosal membrane, and no lymphadenopathy.

**Figure 1:**
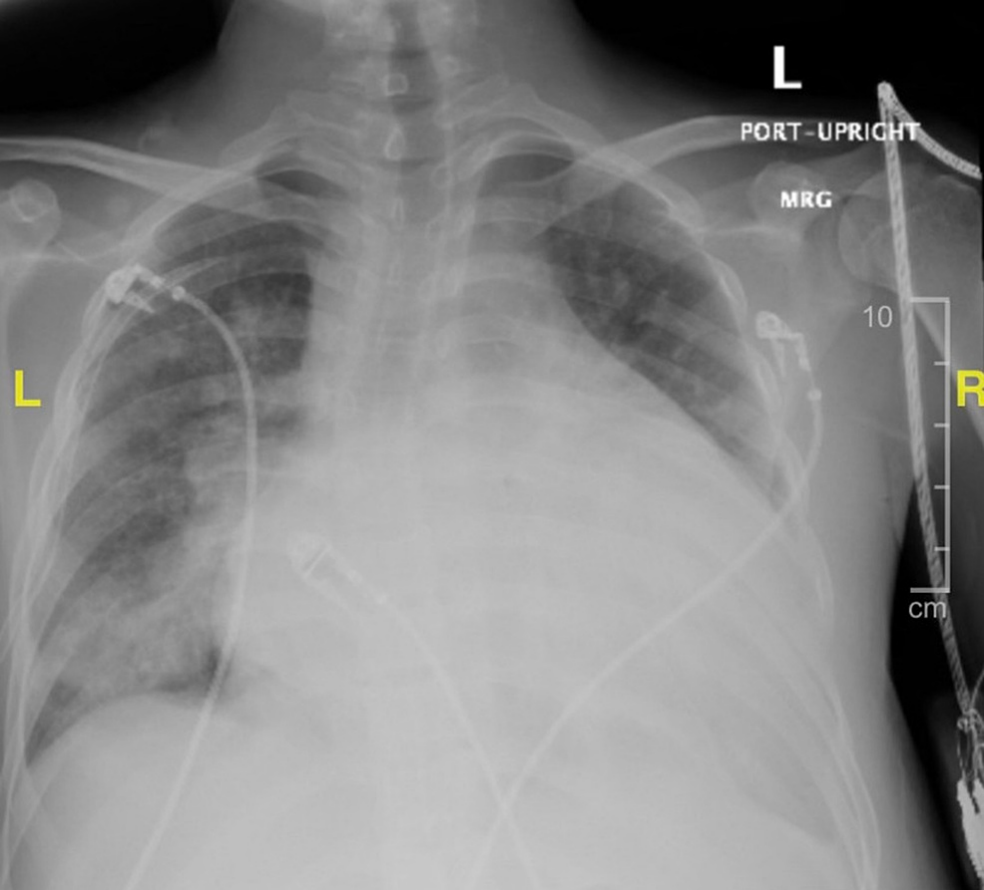
Admission chest X-ray.

Doppler ultrasound study of bilateral lower extremities was negative for deep venous thrombosis. An initial chest X-ray revealed a markedly enlarged cardiac silhouette with vascular congestion, pulmonary consolidation, and small left pleural effusion (Figure 1). Chest computed tomography (CT) without contrast revealed moderate cardiomegaly with moderately large right and small left pleural effusion with pulmonary consolidation, possibly related to infiltrates. CT of the abdomen and pelvis without contrast revealed anasarca without bowel obstructions. Ultrasound of the abdomen showed diffuse ascites, right pleural fluid, and gallbladder sludge. Transthoracic echocardiogram revealed a severely dilated left ventricle with reduced systolic function to approximately 10-15%. Right ventricle systolic pressure was estimated as 86 mmHg, with septum bowing from right to left, consistent with increased right atrial pressure.

The patient was treated with ampicillin/sulbactam, doxycycline, and furosemide before admission to the ICU with a preliminary diagnosis of community-acquired pneumonia and acute decompensated congestive heart failure of unknown etiology resulting in multiorgan failure, including but not limited to congestive hepatopathy and acute kidney injury. Thoracentesis with chest tube placement was performed with the removal of transudative fluid. He was later initiated on vasopressor and inotropic cardiac support.

**Figure 2:**
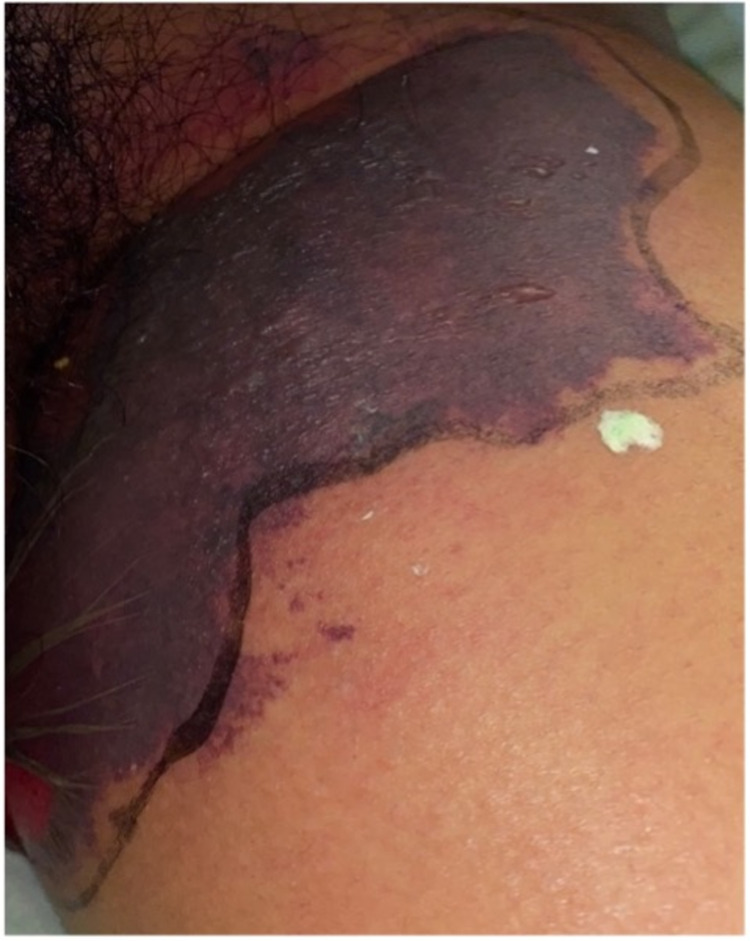
Purpura fulminans on the inner left thigh.

On ICU day two, the rash had evolved from petechia to a significant ecchymosis that was painful and progressively enlarging with bullae formation (Figure 2). Additionally, coagulation studies resulted in abnormal findings: D-dimer level of 25,255 ng/ml, fibrinogen level of 159 mg/dL, prothrombin time (PT) of 52.0 seconds, activated partial thromboplastin time (aPTT) of 59.7 seconds, and international normalized ratio (INR) of 4.5. These were consistent with possible disseminated intravascular coagulation. The rash was diagnosed as PF.

Blood cultures obtained on ICU day three revealed the presence of *K. pneumoniae* in two of two bottles, and the patient was transitioned to a seven-day course of intravenous ceftriaxone treatment. The source of infection was attributed to pneumonia. Serological examination showed negative results for hepatitis B and C, adenovirus, *Bordetella*, *Bordetella pertussis*, HIV, influenza, parainfluenza, respiratory syncytial virus (RSV), rhinovirus, *Trypanosoma cruzi*, legionella, and *Streptococcus pneumoniae*.

Since his admission, hematological markers remained abnormal, with mild improvement over the days. The patient ultimately required transfusion of three units of platelets as his platelet count reached a critical value of 7 × 103/µL.

The cardiology specialist recommended transferring the patient to a different facility for possible cardiac magnetic resonance imaging and biopsy because the cause of congestive heart failure still remained unanswered. However, before the transfer, the patient was urged by his family members to leave the hospital against medical advice. After three days, we were informed of his death at home.

## Discussion

The diagnosis of PF was first documented and described in 1884 as a syndrome of extensive purpura and ecchymosis in critically ill patients in the setting of acute or convalescent infection [4]. Since then, PF has remained a rare, life-threatening condition characterized by concomitant disseminated intravascular coagulation, extensive tissue thrombosis, and hemorrhagic skin necrosis, among other nonspecific symptoms. Most articles on PF are generally case reports or series.

PF is classified into three distinct and broad categories, including neonatal, postinfectious, and acute infectious [2-4]. Neonatal PF is associated with a hereditary deficiency of hematological factors proteins S and C and antithrombin III [2]. This deficiency is due to homozygous or compound heterozygous mutations in *PROC* and *PROS1* genes, as these genes are responsible for encoding proteins C and S, respectively [6]. A lack or deficiency of these proteins results in impeding activation of coagulation in the vasculature. As the term neonatal implies, it manifests very early in life, and treatment is aimed at these deficiencies. Gangrene occurs commonly in the male genitalia, and skin necrosis occurs around the lower and upper limbs.

The postinfectious presentation of PF is often mislabeled as “idiopathic” because it occurs seven to 10 days after infection that is often minor. It is believed to result from acquired autoantibodies against the proteins C and S [7]. The most common infections associated with this etiology are those caused by the varicella virus and *Streptococcus* species of bacteria [8]. This etiology is associated with a lower mortality rate of approximately 15%, with the majority of patients experiencing spontaneous resolution of the autoantibodies by three months [2].

Acute infectious PF is the most common type. Similar to the other etiologies, it is associated with deficiencies of protein C and other hematological proteins. The pathophysiology involves a disruption of coagulation homeostasis resulting from infection by endotoxin-producing gram-negative bacteria as observed in our case with *K. pneumoniae* infection. Along with the resulting inflammatory state, these endotoxins induce the consumption of proteins C and S and antithrombin III, creating a procoagulative state that causes dermal vessel coagulation [4,5]. Infections with gram-positive, anaerobic organisms or viruses [4] have also been documented. Table 2 shows a list of known organisms causing PF, with the meningococcal disease being linked to the largest number of cases [2].

**Table 2:** Infectious causes of purpura fulminans.

Infectious causes of purpura fulminans [2]	
Bacterial	Neisseria meningitidis, Streptococcus pneumoniae, Streptococcus pyogenes, Haemophilus influenzae, Staphylococcus aureus, Leptospira, Enterococcus faecalis, Klebsiella pneumoniae, Escherichia coli, Pseudomonas aeruginosa, Rickettsia rickettsii, Vibrio parahaemolyticus, and Proteus mirabilis
Protozoal	Plasmodium falciparum
Viral	Varicella, zoster, and rubeola

As observed in our young patient, the skin lesion may present early as petechia that rapidly becomes confluent and then progresses into larger ecchymoses. Later in the course, hemorrhagic bullae may form, contributing to the classic hard eschar formation in PF. Laboratory findings associated with PF include prolonged coagulation time, decreased fibrinogen level, elevated D-dimer level, abnormalities in protein C function, and thrombocytopenia [2,5]. Thrombotic complications can even worsen after the clearance of the culprit organism using antibiotics [5]. This situation was observed in our patient after days of supportive care and intravenous ceftriaxone treatment, where we found some, but not complete, improvement in clinical and laboratory markers. This made it clearer that our patient was affected by an acute case of PF in the setting of *K. pneumoniae* septic bacteremia.

In 2020, Nguyen et al. [5] published a case report where only two other cases of PF caused by *K. pneumoniae* were found in the literature. To our knowledge, and after an extensive literature search, the present report makes a total of four cases, indicating the extremely rare presentation of this condition.

As mentioned earlier, PF and all the associated pathophysiological changes comprise a life- and limb-threatening disease with high mortality and significant long-term morbidity in survivors. Patients require intensive care management with prompt recognition and immediate treatment of the underlying cause along with supportive measures of the ongoing multiorgan abnormalities to prevent permanent disability and death. There is limited evidence to guide management, and describing an outline of such management would be out of the scope of this paper. Probably in the future, when there is sufficient evidence, a more tailored understanding and approach to management would become available.

## Conclusions

Considering that PF is a rare finding among the sickest of patients in the critical care setting, we believe that it is important that clinicians should be aware of this diagnosis and have a high index of suspicion when this dermatological finding is paired with disseminated intravascular coagulation and thrombocytopenia. A delay in diagnosis can result in insignificant adverse clinical consequences to the patient, such as loss of limbs and death. We have described a complex case that provided our clinical team to work with, and although a few clinical questions had remained unanswered, we believe that this information could be beneficial to others and potentially have a positive impact on the lives of patients for whom we provide medical care.
